# Impact of stress hyperglycemia ratio, derived from glycated albumin or hemoglobin A1c, on mortality among ST-segment elevation myocardial infarction patients

**DOI:** 10.1186/s12933-023-02061-6

**Published:** 2023-12-06

**Authors:** Wang Liao, Yuwen Chen, Qiyue Gao, Rongrong Gan, Ming Li, Zhenliang Liu, Jiasheng Liang, Henghua Cui, Kaida Ren, Yabin Liu, Zhengdong Wang, Jun Jiang, Qucheng Wei

**Affiliations:** 1 Department of Cardiology, First People’s Hospital of Yulin, Yulin, China; 2 Department of Cardiology, Second Affiliated Hospital, Zhejiang University School of Medicine, Hangzhou, China

**Keywords:** Stress-induced hyperglycemia, ST-segment elevation myocardial infarction, Glycated albumin, Glycated hemoglobin A1c, Prognosis

## Abstract

**Background:**

Stress hyperglycemia ratio (SHR), associated with adverse outcomes in patients with ST-segment elevation myocardial infarction (STEMI), has several definitions. This study aims to assess the prognostic value of SHR, derived from hemoglobin A1c (HbA1c) or glycated albumin (GA), to mortality.

**Methods:**

The study comprised 1,643 STEMI patients who underwent percutaneous coronary intervention (PCI) in two centers. SHR1 was calculated using fasting blood glucose (FBG)/GA, while SHR2 was calculated using the formula FBG/(1.59*HbA1c-2.59). The primary endpoints were in-hospital death and all-cause mortality, with a median follow-up duration of 1.56 years.

**Results:**

Higher SHR1 and SHR2 values are associated with increased risks of in-hospital death and all-cause mortality. Each standard deviation increase in SHR1 corresponded to a 39% and 22% escalation in in-hospital death and all-cause mortality, respectively. The respective increases for SHR2 were 51% and 26%. Further examinations validated these relationships as linear. Additionally, the areas under the curve (AUC) for in-hospital death were not significantly different between SHR1 and SHR2 (p > 0.05). Incorporating SHR1 or SHR2 into the base model significantly improved the discrimination and risk reclassification for in-hospital and all-cause mortality. A subgroup analysis revealed that the effects of SHR1 and SHR2 were more pronounced in patients with hypercholesteremia.

**Conclusion:**

SHR1 and SHR2 have emerged as robust and independent prognostic markers for STEMI patients undergoing PCI. The SHR calculation based on either HbA1c or GA can provide additional predictive value for mortality beyond traditional risk factors, helping to identify high-risk STEMI patients.

**Supplementary Information:**

The online version contains supplementary material available at 10.1186/s12933-023-02061-6.

## Introduction

Cardiovascular disease, is a leading cause of morbidity and mortality globally and frequently presents clinically as acute myocardial infarction (AMI) for the first time [1, 2]. Despite a relative decrease, ST-segment elevation myocardial infarction (STEMI) remains approximately 40% of all AMI presentations [3]. The STEMI mortality rate has been reduced due to advancements in percutaneous coronary intervention (PCI) and pharmaceutical therapies. However, this decrease has reached a plateau, and the mortality rate remains high [4].

Stress hyperglycemia ratio (SHR), a novel indicator of stress-induced hyperglycemia status, pertains to the temporary spike in blood glucose levels in critically ill patients [5–7]. Previous studies have suggested that SHR is linked to a poor prognosis in acute coronary syndrome (ACS) patients [8–10]. However, these studies predominantly concentrated on unstable angina and non-STEMI [8] or simply evaluated the association between SHR and short-term mortality [9, 10]. Furthermore, the literature reveals varying definitions of SHR, dictated by different average glucose level formulas. Hemoglobin A1c (HbA1c) is typically used to calculate this average. A recent study has revealed that glycated albumin (GA), a marker reflecting 2–3 weeks glucose levels, may provide a more accurate calculation [11, 12]. The association between diverse SHR definitions based on HbA1c or GA and the mortality risk associated with STEMI patients undergoing PCI remains unexplored, thus necessitating further investigation.

Therefore, our study’s objective is to assess the prognostic value of different definitions of SHR, calculated on either HbA1c or GA, regarding in-hospital death and long-term mortality in STEMI patients who underwent PCI.

## Methods

### Study design and population

This retrospective cohort study was conducted in two tertiary academic hospitals: The Second Affiliated Hospital of Zhejiang University School of Medicine and the First People's Hospital of Yulin. A total of 1,643 patients who underwent emergency PCI for STEMI between January 1, 2016, and December 31, 2021, were included in the study. The diagnosis criteria of STEMI was established based on definitive clinical signs of myocardial ischemia and novel ischemic alterations on the electrocardiogram, specifically novel ST-segment elevation in two sequential leads (V2-V3 leads ≥ 2 mm in males aged ≥ 40 years, ≥ 2.5 mm in males aged < 40 years, and ≥ 1.5 mm in females irrespective of age; other leads: ≥ 1 mm). Patients were excluded if they: (1) lacked essential laboratory data, such as fasting blood glucose (FBG), GA, and HbA1c; (2) presented with cardiogenic shock; (3) had a life expectancy of less than 9 months; (4) were pregnant or lactating. Coronary angiography and PCI aligned with pertinent guidelines [2, 13]. All patients were administered 300 mg of aspirin, 180 mg of ticagrelor (or 300–600 mg of clopidogrel), and 100 IU/kg of heparin. The PCI was performed via radial or femoral artery access deploying a 6 or 7-Fr catheter, adhering to the standard technique employed by experienced cardiologists. The utilization of glycoprotein IIb/IIIa antagonists was determined by the operator, contingent on the patient's specific clinical state. The study was approved by the local committee. The requirement for written consent was waived because the study was retrospective and non-intrusive.

### Data collection

In this study, demographic and clinical data including ischemic time, age, sex, hypertension, diabetes, hypercholesteremia, atherosclerotic cardiovascular disease (ASCVD), and smoking status, were collected from all participating patients. The diabetes was identified based on historical medical records, documented use of oral hypoglycemic agents or insulin, or an HbA1c level that surpassed 6.5%. Hypercholesterolemia was defined as total cholesterol ≥ 200 mg/dL, high-density lipoprotein (HDL) < 40 mg/dL, low-density lipoprotein (LDL) ≥ 130 mg/dL, or documented use of cholesterol-lowering drugs. Additionally, the gathered clinical data incorporated laboratory parameters, angiographic characteristics, and the treatment regimens implemented at admission and upon discharge. The presence of stenosis exceeding 70% in a minimum of two distinct coronary arteries served as the definition for multivessel coronary disease.

Fasting venous blood samples were obtained within 24 h upon patients’ admission and swiftly transported to the laboratory. GA was expressed as a percentage of total serum albumin to denote GA levels, while HbA1c was a proportion of glycated hemoglobin to total serum hemoglobin. Two measures, namely, SHR1 = FBG (mmol/L)/GA (%) [12] and SHR2 = (FBG (mmol/L))/ (1.59 × HbA1c (%)—2.59) [8], were employed to delineate stress hyperglycemia. The choice of utilizing fasting glucose as the numerator, rather than admission blood glucose (ABG), was anchored in its enhanced prognostic significance in patients with acute cardiovascular disease [14, 15], relative insensitivity to food or other sugar infusions, and limited inter-individual variability [17, 18]. Furthermore, patients were stratified into four distinct subgroups based on the SHR1 quartiles (Q1 ≤ 0.359, Q2 0.359–0.410, Q3 0.410–0.477, and Q4 > 0.477) or SHR2 (Q1 ≤ 0.746, Q2 0.746–0.843, Q3 0.843–0.979, and Q4 > 0.979).

### Follow-up and endpoint

The primary endpoints were defined as in-hospital death and all-cause mortality in STEMI patients treated with PCI. The designated cut-off for long-term follow-up was set for October 1, 2022, ensuring a minimum of 9 months of follow-up. The follow-up data acquisition was facilitated by specially trained independent staff, who collated clinical outcomes via telephonic communication, outpatient consultations, or medical record evaluation. Thus, the data obtained were subject to an adjudication conducted by independent medical experts intentionally kept uninformed regarding the specific study details.

### Statistical analysis

A multivariable logistic regression was employed, and odds ratio (OR) and 95% confidence interval (95% CI) were calculated to evaluate the relationship between SHR1, SHR2, and in-hospital mortality. In contrast, a multivariable Cox regression was utilized to assess the association between SHR1, SHR2, and all-cause mortality, and hazard ratio (HR) and 95% CI were calculated. Three multivariable adjustment models were constructed. Model 1 was adjusted for age and sex, while Model 2 was adjusted for ischemic time, smoking status, hypertension, diabetes, hypercholesterolemia, ASCVD, and estimated glomerular filtration rate (eGFR). Model 3 was adjusted for the culprit vessel and multi-vessel disease. SHR1 and SHR2 were analyzed as continuous and categorical variables (quartiles). The Q1 group was designated as the reference group in the quartile-based analysis. Tests for linear trends were also conducted by including the median value of each category of SHR1 and SHR2 as continuous variables in these models. Restricted cubic splines were employed to comprehensively describe the dose–response curves between SHR1, SHR2, and the outcome. The likelihood ratio test was used to assess the nonlinearity of the risk curves. Subgroup analyses were performed including age, sex, smoking status, hypertension, diabetes, and hypercholesterolemia. The product term p-value was used to evaluate potential interactions. Sensitivity analyses and re-performed logistic and Cox regression analyses were conducted after excluding individuals previously diagnosed with ASCVD.

Furthermore, the receiver operating characteristic (ROC) curve analysis was conducted to evaluate the predictive ability of SHR1 and SHR2, with the respective areas under the curve (AUC) determined. The improvement in model performance, discrimination, and risk stratification after adding SHR1 and SHR2 to the baseline model was quantified using Harrell's C-statistic and C-index, integrated discrimination improvement (IDI), and net reclassification improvement (NRI). A two-sided p < 0.05 was recognized as statistical significance.

## Results

### Baseline characteristics

The present study enrolled a cohort of 1,643 STEMI patients who underwent PCI treatment. The mean ischemic duration was 6.13 h (interquartile range: 4.00–10.78 h), and the patient’s average age was 62.46 ± 12.58 years. The cohort included 1,323 male patients, constituting 80.52% of the total sample. The mean SHR1 and SHR2 values were 0.44 ± 0.14 and 0.89 ± 0.26, respectively. Table 1 and Additional file 2: Table S1 present demographic data stratified into quartiles based on the SHR1 and SHR2 levels. The higher SHR1 quartile (Q4) had a higher proportion of female patients than the lower SHR1 quartile (Q1). This study has an increased prevalence of associated comorbidities, including hypertension, diabetes, and hyperlipidemia. Additionally, Q4 had marked elevation in the TG and LDL levels and a higher incidence of patients with TIMI flow grade 0 and reduced LVEF, than in the Q1 group.

**Table 1:** Baseline demographic and clinical data

	Total	SHR1			
		Q1 (n = 411)	Q2 (n = 409)	Q3 (n = 412)	Q4 (n = 411)
Ischemia time, hours	6.13 (4.00, 10.78)	7.00 (4.23, 11.00)	6.57 (4.00, 11.00)	6.00 (4.00, 10.04)	6.00 (3.84, 10.12)
Age, years	62.46 ± 12.58	65.14 ± 12.39	61.59 ± 12.71	60.64 ± 12.59	62.46 ± 12.24
Sex, male, n (%)	1323 (80.52)	325 (79.08)	335 (81.91)	349 (84.71)	314 (76.40)
Smoking history, n (%)					
Current	787 (47.90)	179 (43.55)	209 (51.10)	222 (53.88)	177 (43.07)
Past	128 (7.79)	31 (7.54)	27 (6.60)	33 (8.01)	37 (9.00)
Medical history, n (%)					
Hypertension	928 (56.48)	228 (55.47)	211 (51.59)	233 (56.55)	256 (62.29)
Diabetes	556 (33.84)	130 (31.63)	106 (25.92)	115 (27.91)	205 (49.88)
Hypercholesteremia	386 (23.49)	72 (17.52)	88 (21.52)	111 (26.94)	115 (27.98)
ASCVD	231 (14.06)	71 (17.27)	52 (12.71)	47 (11.41)	61 (14.84)
Laboratory examinations					
eGFR, ml/min/1.73m^2^	95.68 ± 18.14	92.99 ± 18.02	97.26 ± 17.44	96.89 ± 16.04	95.58 ± 20.53
FBG, mmol/L	7.01 ± 3.05	5.34 ± 1.51	5.97 ± 1.50	6.76 ± 1.92	9.94 ± 4.06
HbA1c, %	6.58 ± 1.54	6.56 ± 1.54	6.30 ± 1.22	6.43 ± 1.38	7.04 ± 1.85
GA, %	16.10 ± 4.43	17.08 ± 4.77	15.52 ± 3.78	15.37 ± 4.35	16.42 ± 4.55
Triglycerides, μmol/L	1.38 (1.02, 2.04)	1.27 (0.95, 1.76)	1.40 (1.06, 2.01)	1.42 (1.01, 2.07)	1.52 (1.06, 2.25)
HDL, μmol/L	1.05 ± 0.25	1.06 ± 0.26	1.05 ± 0.27	1.04 ± 0.23	1.05 ± 0.26
LDL, μmol/L	2.76 ± 0.95	2.57 ± 0.92	2.82 ± 0.92	2.80 ± 0.84	2.85 ± 1.08
LVEF, %	51.45 ± 16.14	52.69 ± 16.83	52.95 ± 15.36	51.65 ± 16.36	48.48 ± 15.61
Procedural information					
Culprit vessel, n (%)					
LM	18 (1.10)	4 (0.97)	1 (0.24)	2 (0.49)	11 (2.68)
LAD	885 (53.86)	223 (54.26)	209 (51.10)	222 (53.88)	231 (56.20)
LCX	177 (10.77)	47 (11.44)	58 (14.18)	38 (9.22)	34 (8.27)
RCA	563 (34.27)	137 (33.33)	141 (34.47)	150 (36.41)	135 (32.85)
Multivessel disease, n (%)					
Two-vessel	407 (24.77)	114 (27.74)	92 (22.49)	102 (24.76)	99 (24.09)
Three-vessel	263 (16.01)	60 (14.60)	66 (16.14)	63 (15.29)	74 (18.00)
TIMI flow, n (%)					
0	665 (40.47)	123 (29.93)	154 (37.65)	193 (46.84)	195 (47.45)
1	43 (2.62)	9 (2.19)	12 (2.93)	11 (2.67)	11 (2.68)
2	86 (5.23)	25 (6.08)	18 (4.40)	22 (5.34)	21 (5.11)
3	849 (51.67)	254 (61.80)	225 (55.01)	186 (45.15)	184 (44.77)
Thrombectomy, n (%)	774 (47.17)	136 (33.17)	182 (44.61)	230 (55.83)	226 (54.99)
Number of stents	1.47 ± 0.80	1.54 ± 0.92	1.47 ± 0.79	1.52 ± 0.80	1.37 ± 0.67
Diameter of stents	3.00 (2.75, 3.50)	3.00 (2.50, 3.50)	3.00 (2.75, 3.50)	3.00 (2.75, 3.50)	3.00 (2.75, 3.50)
Length of stents	31.50 (24.00, 46.00)	32.00 (23.00, 45.25)	32.00 (23.00, 48.00)	32.00 (24.00, 50.00)	30.00 (24.00, 40.00)
Medication, n (%)					
Statins	1598 (97.26)	406 (98.78)	403 (98.53)	404 (98.06)	385 (93.67)
ACEI or ARB	1302 (79.25)	318 (77.37)	338 (82.64)	330 (80.10)	316 (76.89)
β-Blocker	1506 (91.66)	375 (91.24)	382 (93.40)	377 (91.50)	372 (90.51)

Data are means ± SD, median (interquartile range), or n (%)

*ACEI* Angiotensin converting enzyme inhibitors, *ARB* Angiotensin II receptor blockers, *FBS* fasting blood sugar, *GA* glycated albumin, *HbA1c* Glycosylated Hemoglobin, Type A1c, *HDL-C* High density lipoprotein cholesterol, *LAD* Left anterior descending artery, *LCX* Left circumflex artery, *LDL-C* Low density lipoprotein cholesterol, *LM* left main artery, *LVEF* Left ventricular ejection fraction, *RCA* Right coronary artery, *SHR* stress hyperglycemia ratio, *TIMI* Thrombolysis in myocardial infarction

### Association between SHR1, SHR2 and in-hospital death

**Fig. 1:**
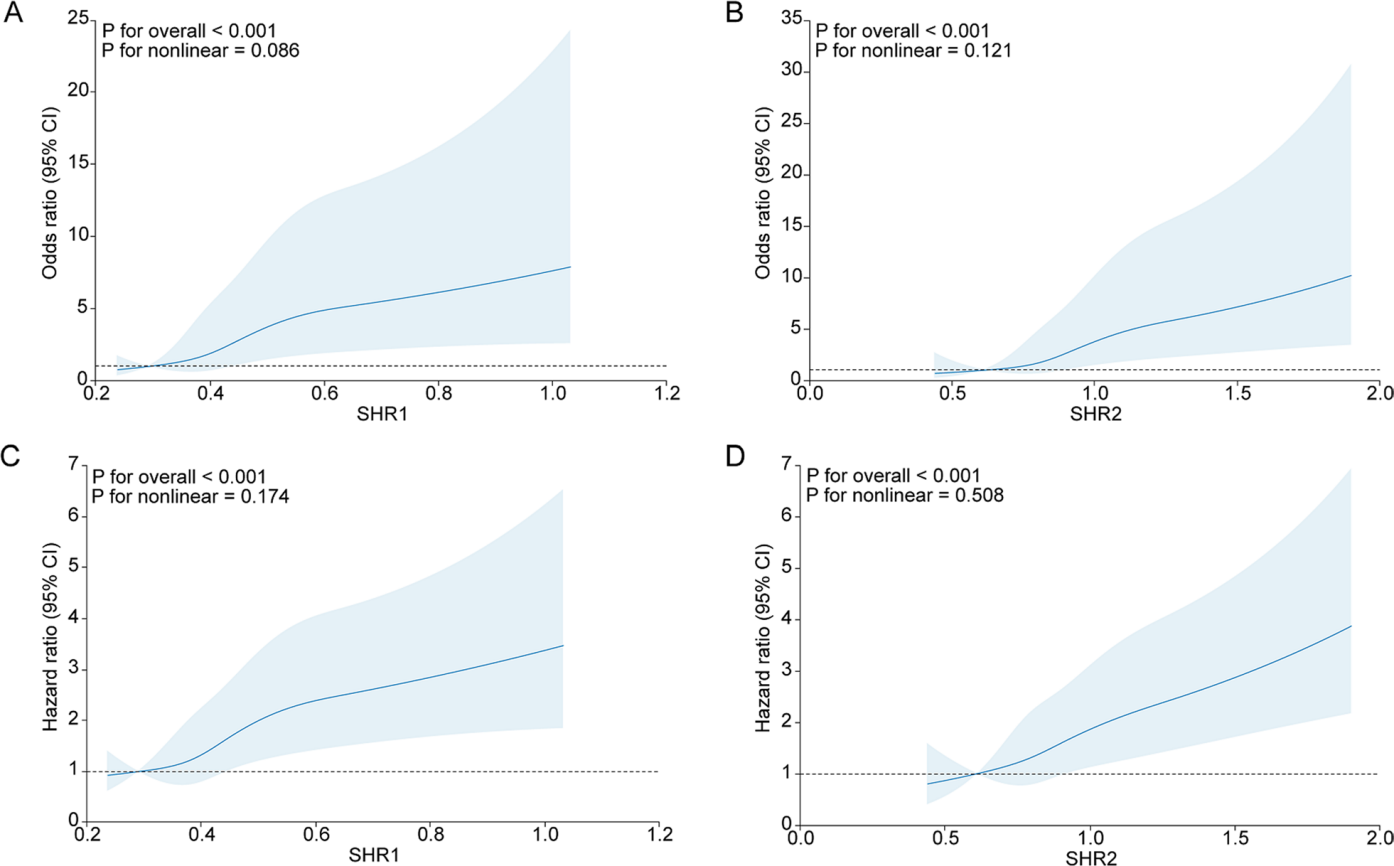
Association of SHR1 and SHR2 with poor prognosis. **A** SHR1 and in-hospital death; **B** SHR2 and in-hospital death; **C** SHR1 and all-cause mortality; **D** SHR2 and all-cause mortality

After adjusting variables, including ischemic time, age, sex, hypertension, diabetes, hypercholesteremia, ASCVD, smoking status, eGFR, culprit vessels, and multivessel disease, a notable correlation between both SHR1 and SHR2 and in-hospital death in STEMI patients was identified. Table 2 illustrates that OR for Q2, Q3, and Q4 was 1.35 (0.49, 3.76), 2.31 (0.91, 5.86), and 4.26 (1.85, 9.83), respectively, denoting a significant trend (p for trend < 0.001) compared to the Q1 group (the reference group). Similarly, Table 2 displays a significant trend of 0.70 (0.25, 2.01), 0.76 (0.28, 2.06), and 3.44 (1.63, 7.26) for Q2, Q3, and Q4, respectively (p for trend < 0.001), compared to OR. Furthermore, Fig. 1 highlights a linear relationship between SHR1, SHR2, and in-hospital mortality after adjusting the confounders previously mentioned (p for nonlinearity > 0.05). A rise in one standard deviation in SHR1 corresponded to a 39% increase in in-hospital mortality risk. A similar result was observed with SHR2, where a rise in one standard deviation indicated a 51% increase in mortality risk.

**Table 2:** Multivariable Logistic and Cox regression analyses for mortality according to SHR1

	SHR1					Per SD increment in SHR1
	≤ 0.359	0.359–0.410	0.410–0.477	> 0.477	*P*_trend_	
In-hospital death						
Model 1	Reference	1.18 (0.43, 3.18)	2.04 (0.83, 5.01)	4.83 (2.18, 10.72)	< 0.001	1.52 (1.30, 1.77)
Model 2	Reference	1.43 (0.52, 3.95)	2.50 (0.98, 6.33)	4.88 (2.13, 11.15)	< 0.001	1.43 (1.21, 1.68)
Model 3	Reference	1.35 (0.49, 3.76)	2.31 (0.91, 5.86)	4.26 (1.85, 9.83)	< 0.001	1.39 (1.17, 1.66)
All-cause mortality						
Model 1	Reference	0.83 (0.48, 1.43)	1.19 (0.71, 1.97)	2.34 (1.51, 3.64)	< 0.001	1.28 (1.18, 1.38)
Model 2	Reference	0.89 (0.51, 1.55)	1.38 (0.82, 2.33)	2.23 (1.42, 3.51)	< 0.001	1.24 (1.13, 1.35)
Model 3	Reference	0.93 (0.53, 1.61)	1.40 (0.83, 2.35)	2.14 (1.36, 3.37)	< 0.001	1.22 (1.10, 1.34)

Model 1: adjusted for age, sex

Model 2: further adjusted for ischemia time, hypertension, hypercholesterolemia, diabetes, ASCVD, smoking status, eGFR

Model 3: further adjusted for culprit vessel, multivessel lesion

### Association between SHR1, SHR2 and all-cause mortality

A total of 143 patients died during a follow-up period of 2,566 person-years (with a median follow-up of 1.56 years). The Kaplan–Meier curve was shown in Additional file 1: Figure S1. Following adjusting for previously mentioned confounders, a distinct correlation was revealed between SHR1, SHR2, and all-cause mortality in STEMI patients. Table 2 depicts that HR for Q2, Q3, and Q4 groups were 0.93 (0.53, 1.61), 1.40 (0.83, 2.35), and 2.14 (1.36, 3.37), respectively, indicating a statistically significant trend (p for trend < 0.001) compared to the SHR1 Q1 group. Similarly, Table 3 reveals an escalation in the risk of all-cause mortality in proportion to SHR2, with HR for Q2, Q3, and Q4 of 0.87 (0.50, 1.52), 1.17 (0.71, 1.93), and 1.89 (1.21, 2.93), respectively (p for trend < 0.001). The RCS demonstrated a linear association between SHR1, SHR2, and all-cause mortality in patients (p for nonlinearity > 0.05). The risk of all-cause mortality augmented by 22% and 26%, with each unit increase in standard deviation in SHR1 and SHR2, respectively.

**Table 3:** Multivariable Logistic and Cox regression analyses for mortality according to SHR2

	SHR2					Per SD increment in SHR2
	≤ 0.746	0.746–0.843	0.843–0.979	> 0.979	Ptrend	
In-hospital death						
Model 1	Reference	0.62 (0.22, 1.72)	0.74 (0.28, 1.96)	3.95 (1.93, 8.08)	< 0.001	1.63 (1.38, 1.92)
Model 2	Reference	0.73 (0.26, 2.07)	0.81 (0.30, 2.19)	3.98 (1.90, 8.33)	< 0.001	1.53 (1.29, 1.81)
Model 3	Reference	0.70 (0.25, 2.01)	0.76 (0.28, 2.06)	3.44 (1.63, 7.26)	< 0.001	1.51 (1.26, 1.81)
All-cause mortality						
Model 1	Reference	0.73 (0.42, 1.27)	1.09 (0.66, 1.79)	2.11 (1.37, 3.25)	< 0.001	1.33 (1.22, 1.44)
Model 2	Reference	0.88 (0.50, 1.54)	1.23 (0.75, 2.03)	2.04 (1.32, 3.17)	< 0.001	1.29 (1.18, 1.41)
Model 3	Reference	0.87 (0.50, 1.52)	1.17 (0.71, 1.93)	1.89 (1.21, 2.93)	< 0.001	1.26 (1.14, 1.40)

Model 1: adjusted for age, sex

Model 2: further adjusted for ischemia time, hypertension, hypercholesterolemia, diabetes, ASCVD, smoking status, eGFR

Model 3: further adjusted for culprit vessel, multivessel lesion

### Predictive value of SHR1 and SHR2 for mortality

**Fig. 2:**
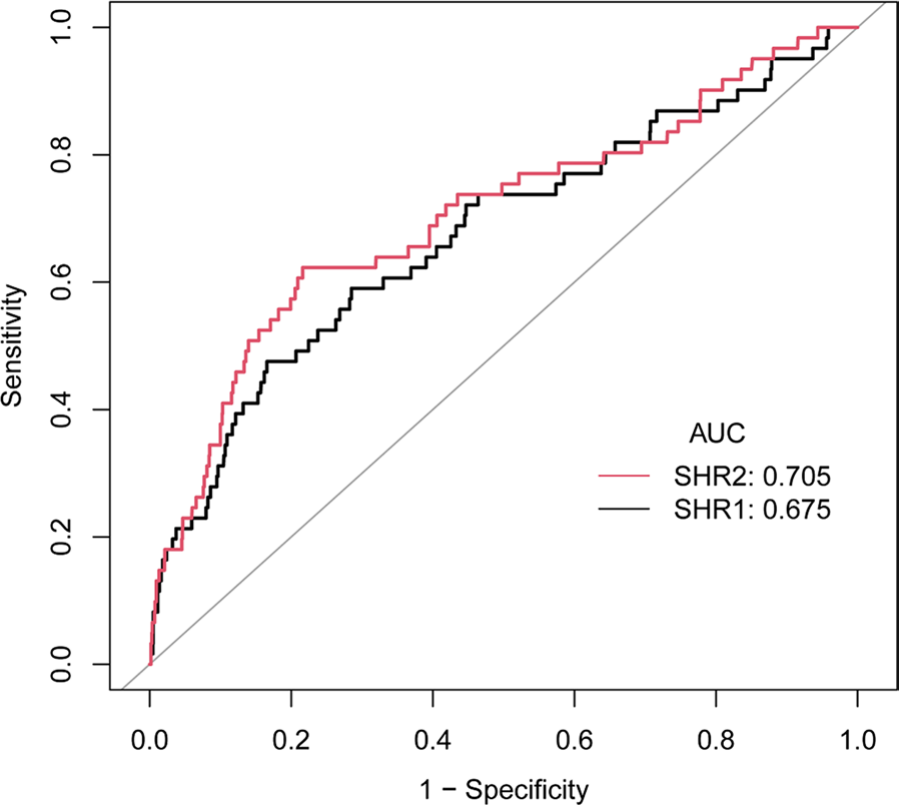
ROC curves of SHR1 and SHR2 to predict the in-hospital death in the overall study population. The AUC of SHR1 was 0.675, 95% CI 0.598–0.752; the AUC of SHR2 was 0.705, 95% CI 0.629–0.782 (p = 0.06)

ROC curve analysis (Fig. 2) demonstrated that SHR1 and SHR2 had a moderate predictive capacity for in-hospital mortality in STEMI patients who had undergone PCI (AUC for SHR1: 0.675, 95% CI 0.598–0.752; AUC for SHR2: 0.705, 95% CI 0.629–0.782). The AUC for SHR1 and SHR2 did not differ significantly (p > 0.05). Furthermore, Table 4 presents that the C-statistic for the base model is 0.783 (95% CI 0.723—0.843). Notably, adding SHR1 to the model significantly enhanced its predictive power, raising the C-statistic to 0.808 (0.747–0.868; p < 0.05). Similarly, incorporating SHR2 into the model led to a significant improvement, elevating the C-statistic to 0.819 (0.761–0.877), thereby indicating an advancement compared to the base model (p < 0.05).

**Table 4:** C-statistics for discrimination ability of different measures of stress hyperglycemia for in-hospital death

	C-Statistic (95% CI)	P value
Established risk factors	0.783 (0.723, 0.843)	reference
Established risk factors + SHR1	0.808 (0.747, 0.868)	0.03
Established risk factors + SHR2	0.819 (0.761, 0.877)	0.01

Established risk factors included age, sex, ischemia time, hypertension, hypercholesterolemia, diabetes, ASCVD, smoking status, eGFR, culprit vessel, multivessel lesion

Introducing SHR1 and SHR2 into the base model notably improved the prediction potential for all-cause mortality. Table 5 indicates that the inclusion of SHR1 raised the C-index from 0.782 (95% CI 0.740–0.825) to 0.793 (95% CI 0.750—0.836), and further inclusion of SHR2 increased it to 0.798 (95% CI 0.756–0.840). After adding SHR1 and SHR2 to the base model, the IDI reported a significant advancement of 1.70% (95% CI 0.20–4.30%, p = 0.007) and 2.10% (95% CI 0.40—5.20%, p < 0.001), respectively. The NRI affirmed significance for SHR2 (21.00%, 95% CI 4.50–32.80%, p = 0.02), but not for SHR1 (11.60%, 95% CI −2.20 to 23.60%, p = 0.13).

**Table 5:** Improvement in discrimination and risk reclassification for all-cause mortality after the addition of SHR1 or SHR2

Model	C-index (95%CI)	IDI (%) (95%CI)	P-value	NRI (%) (95%CI)	P-value
Established risk factors	0.782 (0.740, 0.825)	Ref	Ref	Ref	Ref
Established risk factors + SHR1	0.793 (0.750, 0.836)	1.70 (0.20, 4.30)	0.007	11.60 (−2.20, 23.60)	0.13
Established risk factors + SHR2	0.798 (0.756, 0.840)	2.10 (0.40, 5.20)	< 0.001	21.00 (4.50, 32.80)	0.02

Established risk factors include age, sex, ischemia time, hypertension, hypercholesterolemia, diabetes, ASCVD, smoking status, eGFR, culprit vessel, multivessel lesion

### Subgroup and sensitivity analyses

**Fig. 3:**
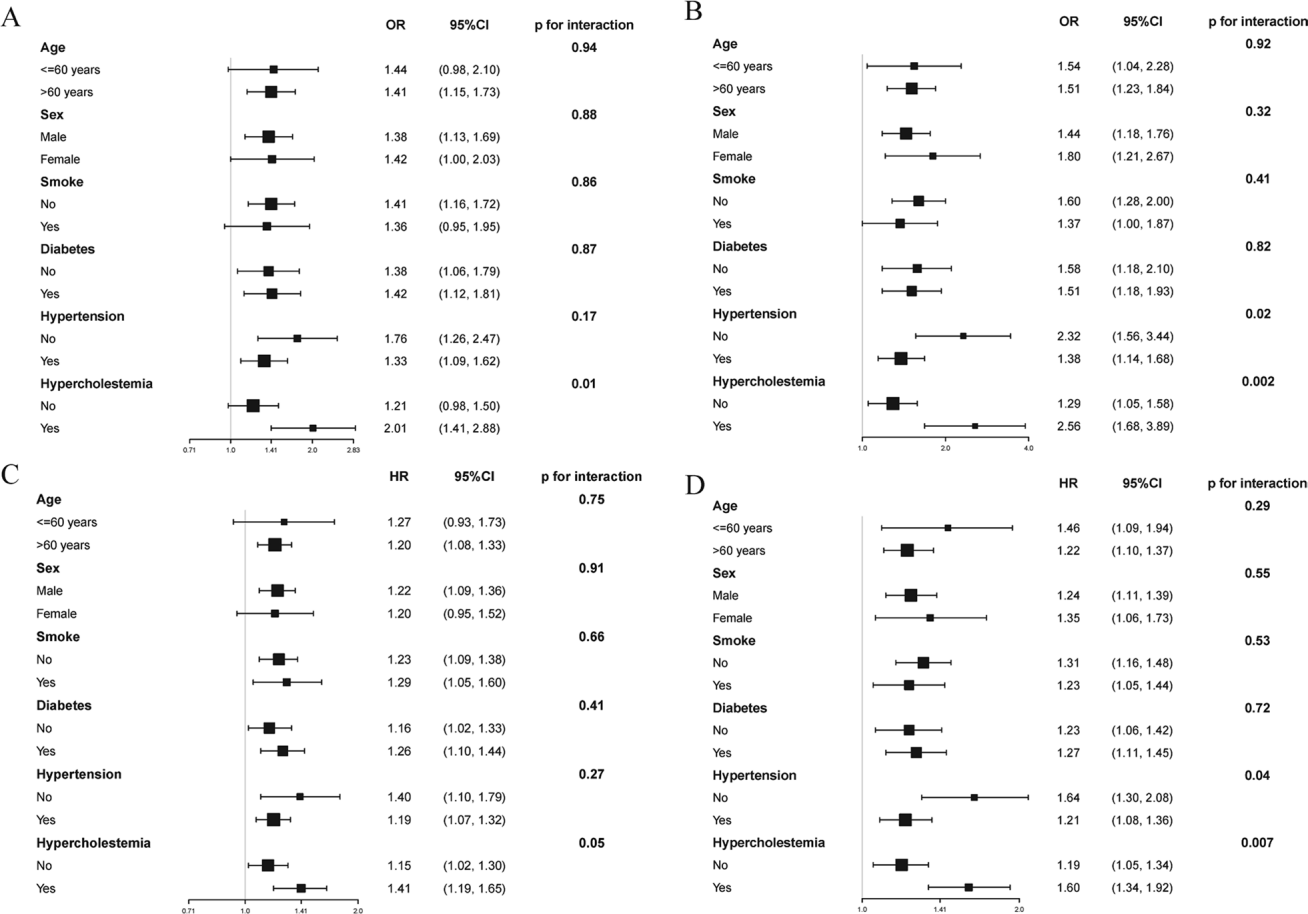
Subgroup analyses of SHR1 and SHR2 in different populations. **A** In-hospital death according to SHR1; **B** In-hospital death according to SHR2; **C** All-cause mortality according to SHR1; **D** All-cause mortality according to SHR2

We conducted subgroup analysis to explore the relationship between SHR1, SHR2, and mortality within diverse subgroups and ascertain the robustness of our results (Fig. 3). We identified a direct correlation between SHR1 and SHR2 and in-hospital mortality, as well as all-cause mortality, upon adjusting confounding variables. Our analysis revealed that the impact of SHR1 and SHR2 was markedly augmented in the patients with hypercholesterolemia (p for interaction ≤ 0.05). Additionally, we excluded patients with a prior diagnosis of ASCVD, and the results were sustained and consistent (Additional file 3: Table S2 and Additional file 4: Table S3). We made additional adjustments to the LVEF and TIMI flow, and the results remained steady and coherent (Additional file 5: Table S4 and Additional file 6: Table S5).

## Discussion

This cohort study identified a robust correlation between mortality in STEMI patients who received PCI treatment and the SHR, calculated using GA or HbA1c. This association remained independent of traditional risk factors, such as age, sex, smoking habits, hypertension, hyperlipidemia, diabetes, and renal impairment. Our findings underscore the role of SHR as a risk factor for the unfavorable prognosis in STEMI patients. Our analysis revealed a linear relationship, indicating that incorporating SHR1 or SHR2, besides traditional risk factors, improved the prediction of in-hospital and all-cause mortality in STEMI patients. Additionally, our study included various subgroup and sensitivity analyses; the outcomes were stable. We discovered that the effects of SHR1 and SHR2 were enhanced in patients with hypercholesteremia. To our knowledge, this is the first study to compare the predictive value between different calculations of SHR among STEMI patients treated with PCI.

### Mechanism and detrimental effects of stress hyperglycemia

Stress-induced hyperglycemia has been activated by the hypothalamic–pituitary–adrenal axis, elevating cortisol and adrenaline secretion and exacerbating the hyperglycemia state [16]. Furthermore, this condition has been associated with the upregulation of pro-inflammatory cytokines, including interleukin-1, interleukin-6, and tumor necrosis factor-α. These cytokines may impair insulin secretion and augment insulin resistance [17–19]. These inflammatory mediators have been implicated in the worsening of atherosclerosis [20, 21]. Additionally, stress-induced hyperglycemia may contribute to increased thrombogenic activity, culminating in a hypercoagulable state [22, 23]. Previous studies exhibited that higher SHR was associated with a larger thrombus burden and lower TIMI flow grade during angiography [24, 25].

Our study's conclusions partially align with those of certain previous investigations. Previous studies have recognized stress-induced hyperglycemia as an adverse prognostic risk factor in ACS patients [8, 9, 11, 26, 27]. This study determined that the SHR, derived from either HbA1c or GA, was associated with in-hospital mortality and long-term adverse prognosis in STEMI patients. The possibility of a non-linear relationship between SHR and adverse prognosis has been debated in prior studies. Yang et al. enrolled 5,562 ACS patients, 410 of whom had STEMI, and identified a U-shaped correlation between SHR, based on HbA1c, and adverse prognosis [8]. Similarly, Wang et al. identified a U-shaped relationship between SHR, as determined by GA, and adverse prognosis in 5,190 ACS patients, including 1,320 STEMI cases [11]. Similar results were found in patients with heart failure and diabetes [28]. Conversely, Fang et al.'s found no U-shaped relationship between SHR and in-hospital death among 8,196 patients (3,001 with ACS and the remainder with stable angina [9]. Our analysis discovered a linear association between SHR, calculated from HbA1c or GA, and both in-hospital and all-cause mortality. This discrepancy could be attributed to earlier studies using ABG levels for SHR calculation, which various factors may influence. Another plausible explanation is the predominance of unstable angina patients in the cohorts of previous investigations, whereas our study solely encompassed STEMI patients.

### Different definitions of stress hyperglycemia

Several indices are employed to assess the extent of stress-induced hyperglycemia, including ABG, FBG, and SHR. However, ABG and FBG are deemed insufficient to accurately represent stress-induced hyperglycemia because baseline blood glucose levels influence them. In contrast, the SHR, calculated by dividing the current blood glucose level by the average blood glucose level, may more precisely reflect stress-induced hyperglycemia states. Nevertheless, a consensus on the optimal method to determine average blood glucose levels is lacking. However, earlier studies frequently utilized HbA1c to estimate past blood glucose levels. Recent investigations have suggested that GA may provide a superior measure due to its independence from renal insufficiency and anemia [29, 30]. To our knowledge, our study is the first to evaluate the impact of different SHR calculations on prognostic prediction. Our study outcomes reveal that the prognostic accuracy of SHR1 for in-hospital death was comparable to SHR2 via a comparative evaluation of ROC curves. Additionally, the discrimination capabilities of SHR1 and SHR2 for in-hospital death and all-cause mortality were significantly superior to the base model, including traditional risk factors.

Previous studies have debated the influence of diabetes status on the relationship between SHR and mortality. Wang et al. demonstrated that SHR exhibited no substantial connection with all-cause mortality and cardiovascular death among ACS patients in a non-diabetic population [11]. Conversely, Cui et al. found a significant association between SHR and in-hospital death among myocardial infarction individuals, regardless of their diabetes status [9]. Our study discovered that SHR, determined by either HbA1c or GA, was associated with in-hospital mortality and all-cause mortality in diabetic and non-diabetic cohorts. Furthermore, we noted a significant enhancement in the association between SHR and mortality in hypercholesterolemia patients, indicating a potential interactive effect between hypercholesterolemia and SHR. Previous studies have suggested that insulin resistance may precipitate lipid irregularities, identifiable by reduced HDL levels [31, 32]. Inversely, HDL can influence glucose metabolism, with a correlation between higher HDL and lower blood glucose levels [33–36]. The stress-induced hyperglycemia and lipid abnormalities may provoke endothelial dysfunction, consequently elevating the risk of cardiovascular events.

### Predictive value of SHR

Currently, the most used prognostic model for STEMI patients is the TIMI risk score [37, 38]. However, the TIMI risk score primarily focuses on cardiovascular-related risk factors and does not incorporate important metabolic factors into the scoring system. Previous studies have demonstrated that the predictive ability of the TIMI risk score is generally limited [39, 40]. Some studies have suggested that adding SHR to the risk score may be useful for early risk stratification [10, 41, 42]. This study found that SHR remained an independent risk factor for in-hospital death and all-cause mortality in STEMI patients even after adjusting for traditional risk factors. Moreover, FBG, HbA1c, and GA are easily obtained in clinic settings at low cost. This study discovered that integrating the SHR and the traditional risk factors improved the predictive ability of short- and long-term adverse outcomes in STEMI patients. This study suggests that the SHR should be included as an independent risk factor in constructing new prognostic models for STEMI patients.

Many guidelines exist to regulate blood glucose in critically ill patients, signifying the persisting variations in stress hyperglycemia management [43–45]. Despite the efficacy of numerous medications in glucose control, the advantages of hypoglycemic intervention in stress hyperglycemia patients remain debatable [46–52]. However, these studies primarily concentrated on AMI patients with diabetes, not on stress-induced hyperglycemia. Future research is necessitated to establish whether hypoglycemic therapy could enhance the prognosis in AMI patients with stress-induced hyperglycemia.

### Strengths and limitations

Our study’s primary strength is to compare different SHR calculation methods and their association with the prognosis of STMEI patients. To our knowledge, our study is the first to compare the predicted value of different formulas of SHR on in-hospital death and all-cause mortality. Moreover, our study analyzed short- and long-term outcomes, yielding consistent results. Additionally, we performed subgroup and sensitivity analyses to support the robustness of our findings further. However, several limitations should be acknowledged. First, the study was executed across two tertiary academic hospitals and included a comparatively limited sample size, consisting entirely of Asian patients, necessitating a prudent result interpretation. Second, the inherent design of the cohort study precludes inference of a causal relationship within our investigation; verification of these outcomes requires additional prospective studies. Lastly, the lack of data regarding the hypoglycemic therapy application during the monitoring period inhibits our ability to gauge its impact on patients with STEMI.

## Conclusion

Our study highlighted association between SHR, calculated from either GA or HbA1c and in-hospital death and all-cause mortality in STEMI patients who received PCI. The discriminating ability of SHR derived from GA was similar to that derived from HbA1c. In summary, SHR based on either GA or HbA1c proved an effective stratification marker for risk assessment in STEMI patients.

### Supplementary Information

**Additional file 1: Figure S1.** Kaplan–Meier curve for SHR1 (A) and SHR2 (B).

**Additional file 2: Table S1.** Baseline demographic and clinical data

**Additional file 3: Table S2.** Regression analyses for mortality according to SHR1 after excluding participants with ASCVD

**Additional file 4: Table S3.** Regression analyses for mortality according to SHR2 after excluding participants with ASCVD

**Additional file 5: Table S4.** Multivariable Logistic and Cox regression analyses for mortality according to SHR1

**Additional file 6: Table S5.** Multivariable Logistic and Cox regression analyses for mortality according to SHR2

## Data Availability

A reasonable request to the corresponding author will provide access to the data utilized in this study.

## Abbreviations

ABG: Admission blood glucose
ACS: Acute coronary syndrome
AMI: Acute myocardial infarction
ASCVD: Atherosclerotic cardiovascular disease
AUC: Areas under the curve
eGFR: Estimated glomerular filtration rate
FBG: Fasting blood glucose
GA: Glycated albumin
HbA1c: Hemoglobin A1c
HDL: High-density lipoprotein
IDI: Integrated discrimination improvement
LDL: Low-density lipoprotein
NRI: Net reclassification improvement
PCI: Percutaneous coronary intervention
ROC: Receiver operating characteristic
SHR: Stress hyperglycemia ratio
STEMI: ST-segment elevation myocardial infarction
