# Supplementary figures and images for: Impact of stress hyperglycemia ratio, derived from glycated albumin or hemoglobin A1c, on mortality among ST-segment elevation myocardial infarction patients
Source: Cardiovasc Diabetol. 2023 Dec 6;22:334. doi: 10.1186/s12933-023-02061-6 (PMC10701979; doi:10.1186/s12933-023-02061-6)

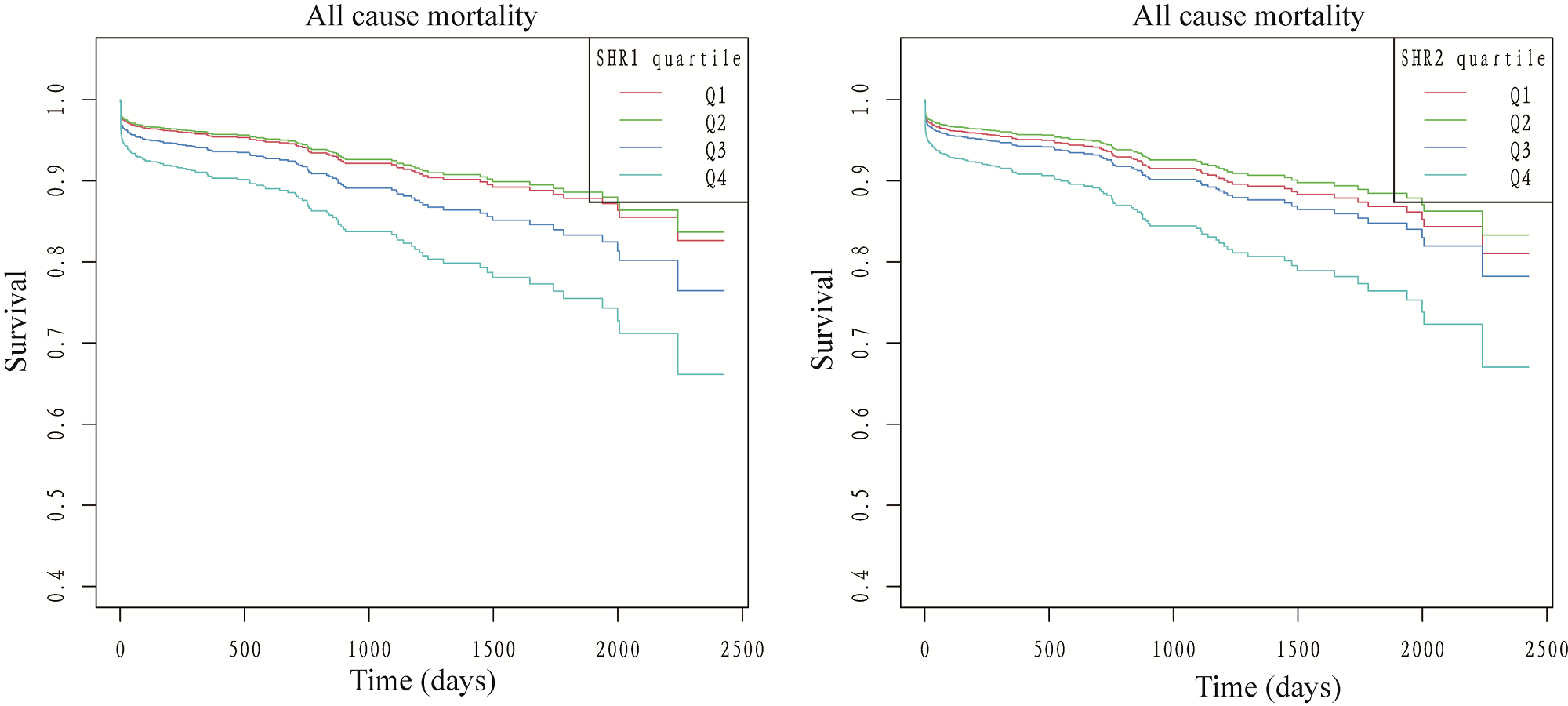

Supplement: Supplementary file 1 — Additional file 1: Figure S1. Kaplan–Meier curve for SHR1 (A) and SHR2 (B). [file 12933_2023_2061_MOESM1_ESM.tif]
