# Supplementary material for: Impact of stress hyperglycemia ratio, derived from glycated albumin or hemoglobin A1c, on mortality among ST-segment elevation myocardial infarction patients
Source: Cardiovasc Diabetol. 2023 Dec 6;22:334. doi: 10.1186/s12933-023-02061-6 (PMC10701979; doi:10.1186/s12933-023-02061-6)
Supplement: Supplementary file 2 — Additional file 2: Table S1. Baseline demographic and clinical data [file 12933_2023_2061_MOESM2_ESM.docx]

**Additional Table 1.** Baseline demographic and clinical data

|  | SHR2 | | | |
| --- | --- | --- | --- | --- |
|  | Q1 (n=411) | Q2 (n=410) | Q3 (n=411) | Q4 (n=411) |
| Ischemia time, hours | 7.00(4.00, 11.00) | 6.50(4.04, 10.14) | 6.40(4.00, 11.00) | 5.92(3.99, 10.00) |
| Age, years | 62.75±12.77 | 61.52±13.08 | 61.75±12.18 | 63.79±12.21 |
| Sex, male, n(%) | 337(82) | 328(80) | 346(84.18) | 312(75.91) |
| Smoking history, n(%) |  |  |  |  |
| Current | 209(50.85) | 202(49.27) | 203(49.39) | 173(42.09) |
| Past | 28(6.81) | 31(7.56) | 36(8.76) | 33(8.03) |
| Medical history, n(%) |  |  |  |  |
| Hypertension | 235(57.18) | 220(53.66) | 223(54.26) | 250(60.83) |
| Diabetes | 161(39.17) | 103(25.12) | 110(26.76) | 182(44.28) |
| Hypercholesteremia | 108(26.28) | 101(24.63) | 82(19.95) | 95(23.11) |
| ASCVD | 64(15.57) | 56(13.66) | 57(13.87) | 54(13.14) |
| Laboratory examinations |  |  |  |  |
| eGFR, ml/min/1.73m^2^ | 95.26±17.19 | 98.14±18.35 | 96.70±18.15 | 92.63±18.46 |
| FBG, mmol/L | 5.43±1.60 | 5.89±1.51 | 6.91±2.26 | 9.79±3.96 |
| HbA1c, % | 6.89±1.67 | 6.29±1.21 | 6.44±1.56 | 6.71±1.61 |
| GA, % | 16.30±4.59 | 15.21±3.83 | 15.95±4.30 | 16.92±4.79 |
| Triglycerides, μmol/L | 1.35(1.01, 2.04) | 1.39(1.05, 2.04) | 1.38(0.98, 1.97) | 1.41(1.04, 2.16) |
| HDL, μmol/L | 1.03±0.25 | 1.06±0.23 | 1.06±0.26 | 1.06±0.26 |
| LDL, μmol/L | 2.67±0.88 | 2.80±0.98 | 2.77±0.93 | 2.81±1.00 |
| LVEF, % | 53.78±16.17 | 54.07±15.37 | 51.11±15.15 | 46.79±16.80 |
| Procedural information |  |  |  |  |
| Culprit vessel, n(%) |  |  |  |  |
| LM | 3(0.73) | 2(0.49) | 3(0.73) | 10(2.43) |
| LAD | 208(50.61) | 216(52.68) | 226(54.99) | 235(57.18) |
| LCX | 63(15.33) | 49(11.95) | 35(8.52) | 30(7.30) |
| RCA | 137(33.33) | 143(34.88) | 147(35.77) | 136(33.09) |
| Multivessel disease, n(%) |  |  |  |  |
| Two-vessel | 109(26.52) | 104(25.37) | 103(25.06) | 91(22.14) |
| Three-vessel | 64(15.57) | 6(14.88) | 62(15.09) | 76(18.49) |
| TIMI flow, n(%) |  |  |  |  |
| 0 | 158(38.44) | 168(40.98) | 164(39.90) | 175(42.58) |
| 1 | 9(2.19) | 12(2.93) | 12(2.92) | 10(2.43) |
| 2 | 22(5.35) | 26(6.34) | 18(4.38) | 20(4.87) |
| 3 | 222(54.01) | 204(49.76) | 217(52.80) | 206(50.12) |
| Thrombectomy, n(%) | 161(39.17) | 192(47.06) | 208(50.61) | 213(51.82) |
| Number of stents | 1.45±0.85 | 1.49±0.85 | 1.49±0.75 | 1.47±0.77 |
| Diameter of stents | 3.00(2.50, 3.50) | 3.00(2.75, 3.50) | 3.00(2.75, 3.50) | 3.00(2.75, 3.50) |
| Length of stents | 30.00(23.00, 41.00) | 32.00(24.00, 48.00) | 33.00(24.00, 48.00) | 30.50(24.00, 45.00) |
| Medication, n(%) |  |  |  |  |
| Statins | 407(99.03) | 402(98.05) | 403(98.05) | 386(93.92) |
| ACEI or ARB | 334(81.27) | 325(79.27) | 341(82.97) | 302(73.48) |
| β-Blocker | 381(92.70) | 375(91.46) | 381(92.70) | 369(89.78) |

Data are means ± SD, median (interquartile range), or n (%).

Abbreviations: ACEI, Angiotensin converting enzyme inhibitors; ARB, Angiotensin II receptor blockers; FBS, fasting blood sugar; GA: glycated albumin; HbA1c, Glycosylated Hemoglobin, Type A1c; HDL-C, High density lipoprotein cholesterol; LAD, Left anterior descending artery; LCX, Left circumflex artery; LDL-C, Low density lipoprotein cholesterol; LM, left main artery; LVEF, Left ventricular ejection fraction; RCA, Right coronary artery; SHR, stress hyperglycemia ratio; TIMI, Thrombolysis in myocardial infarction.
