# Supplementary material for: Impact of stress hyperglycemia ratio, derived from glycated albumin or hemoglobin A1c, on mortality among ST-segment elevation myocardial infarction patients
Source: Cardiovasc Diabetol. 2023 Dec 6;22:334. doi: 10.1186/s12933-023-02061-6 (PMC10701979; doi:10.1186/s12933-023-02061-6)
Supplement: Supplementary file 3 — Additional file 3: Table S2. Regression analyses for mortality according to SHR1 after excluding participants with ASCVD [file 12933_2023_2061_MOESM3_ESM.docx]

**Additional Table 2.** Regression analyses for mortality according to SHR1 after excluding participants with ASCVD

|  | SHR1 | | | | | Per SD increment in SHR1 |
| --- | --- | --- | --- | --- | --- | --- |
|  | ≤0.361 | 0.361-0.412 | 0.412-0.475 | >0.475 | *P*_trend_ |  |
| In-hospital death |  |  |  |  |  |  |
| Model 1 | Reference | 1.62(0.51, 5.18) | 2.47(0.83, 7.37) | 5.08(1.89, 13.69) | <0.001 | 1.56(1.30, 1.88) |
| Model 2 | Reference | 1.74(0.53, 5.71) | 2.63(0.86, 8.06) | 4.26(1.54, 11.81) | 0.002 | 1.43(1.17, 1.75) |
| Model 3 | Reference | 1.78(0.54, 5.92) | 2.61(0.85, 8.02) | 4.16(1.49, 11.61) | 0.003 | 1.44(1.18, 1.77) |
| All-cause mortality |  |  |  |  |  |  |
| Model 1 | Reference | 0.92(0.50, 1.68) | 1.23(0.70, 2.16) | 2.19(1.33, 3.62) | <0.001 | 1.41(1.26, 1.58) |
| Model 2 | Reference | 0.88(0.48, 1.61) | 1.31(0.74, 2.32) | 1.83(1.09, 3.05) | 0.006 | 1.29(1.13, 1.46) |
| Model 3 | Reference | 0.95(0.51, 1.74) | 1.37(0.77, 2.43) | 1.80(1.08, 3.01) | 0.01 | 1.28(1.13, 1.46) |

Model 1: adjusted for age, sex.

Model 2: further adjusted for ischemia time, hypertension, hypercholesterolemia, diabetes, smoking status, eGFR.

Model 3: further adjusted for culprit vessel, multivessel lesion.
