# Supplementary material for: Impact of stress hyperglycemia ratio, derived from glycated albumin or hemoglobin A1c, on mortality among ST-segment elevation myocardial infarction patients
Source: Cardiovasc Diabetol. 2023 Dec 6;22:334. doi: 10.1186/s12933-023-02061-6 (PMC10701979; doi:10.1186/s12933-023-02061-6)
Supplement: Supplementary file 4 — Additional file 4: Table S3. Regression analyses for mortality according to SHR2 after excluding participants with ASCVD [file 12933_2023_2061_MOESM4_ESM.docx]

**Additional Table 3.** Regression analyses for mortality according to SHR2 after excluding participants with ASCVD

|  | SHR2 | | | | | Per SD increment in SHR2 |
| --- | --- | --- | --- | --- | --- | --- |
|  | ≤ |  |  | > | *P*_trend_ |  |
| In-hospital death |  |  |  |  |  |  |
| Model 1 | Reference | 0.38(0.10, 1.46) | 0.65(0.21, 2.02) | 3.49(1.56, 7.80) | <0.001 | 1.74(1.44, 2.11) |
| Model 2 | Reference | 0.50(0.13, 1.96) | 0.70(0.22, 2.24) | 3.25(1.42, 7.45) | <0.001 | 1.60(1.31, 1.95) |
| Model 3 | Reference | 0.49(0.13, 1.93) | 0.69(0.22, 2.21) | 3.05(1.32, 7.05) | 0.002 | 1.62(1.33, 1.98) |
| All-cause mortality |  |  |  |  |  |  |
| Model 1 | Reference | 0.78(0.42, 1.45) | 1.20(0.70, 2.08) | 1.98(1.21, 3.23) | 0.002 | 1.46(1.31, 1.63) |
| Model 2 | Reference | 0.99(0.53, 1.86) | 1.34(0.77, 2.32) | 1.83(1.11, 3.00) | 0.01 | 1.35(1.20, 1.51) |
| Model 3 | Reference | 0.97(0.52, 1.82) | 1.30(0.75, 2.25) | 1.71(1.04, 1.43) | 0.02 | 1.34(1.19, 1.52) |

Model 1: adjusted for age, sex.

Model 2: further adjusted for ischemia time, hypertension, hypercholesterolemia, diabetes, smoking status, eGFR.

Model 3: further adjusted for culprit vessel, multivessel lesion.
