# Supplementary material for: Impact of stress hyperglycemia ratio, derived from glycated albumin or hemoglobin A1c, on mortality among ST-segment elevation myocardial infarction patients
Source: Cardiovasc Diabetol. 2023 Dec 6;22:334. doi: 10.1186/s12933-023-02061-6 (PMC10701979; doi:10.1186/s12933-023-02061-6)
Supplement: Supplementary file 5 — Additional file 5: Table S4. Multivariable Logistic and Cox regression analyses for mortality according to SHR1 [file 12933_2023_2061_MOESM5_ESM.docx]

**STable 4.** Multivariable Logistic and Cox regression analyses for mortality according to SHR1

|  | SHR1 | | | | | Per SD increment in SHR1 |
| --- | --- | --- | --- | --- | --- | --- |
|  | ≤0.359 | 0.359-0.410 | 0.410-0.477 | >0.477 | *P*_trend_ |  |
| In-hospital death |  |  |  |  |  |  |
| Model 3+LVEF+TIMI | Reference | 1.67(0.58, 4.87) | 1.96(0.73, 5.27) | 3.74(1.55, 9.02) | 0.002 | 1.37(1.14, 1.65) |
| All-cause mortality |  |  |  |  |  |  |
| Model 3+LVEF+TIMI | Reference | 1.03(0.59, 1.81) | 1.27(0.75, 2.15) | 1.79(1.13, 2.86) | 0.009 | 1.20(1.08, 1.34) |

Model 3: adjusted for age, sex, ischemia time, hypertension, hypercholesterolemia, diabetes, ASCVD, smoking status, eGFR, culprit vessel, multivessel lesion.
