# Supplementary material for: Impact of stress hyperglycemia ratio, derived from glycated albumin or hemoglobin A1c, on mortality among ST-segment elevation myocardial infarction patients
Source: Cardiovasc Diabetol. 2023 Dec 6;22:334. doi: 10.1186/s12933-023-02061-6 (PMC10701979; doi:10.1186/s12933-023-02061-6)
Supplement: Supplementary file 6 — Additional file 6: Table S5. Multivariable Logistic and Cox regression analyses for mortality according to SHR2 [file 12933_2023_2061_MOESM6_ESM.docx]

**STable 5.** Multivariable Logistic and Cox regression analyses for mortality according to SHR2

|  | SHR2 | | | | | Per SD increment in SHR2 |
| --- | --- | --- | --- | --- | --- | --- |
|  | ≤0.746 | 0.746-0.843 | 0.843-0.979 | >0.979 | *P*_trend_ |  |
| In-hospital death |  |  |  |  |  |  |
| Model 3+LVEF+TIMI | Reference | 0.68(0.23, 2.04) | 0.63(0.21, 1.85) | 2.77(1.25, 6.13) | 0.002 | 1.49(1.23, 1.81) |
| All-cause mortality |  |  |  |  |  |  |
| Model 3+LVEF+TIMI | Reference | 0.88(0.50, 1.54) | 1.06(0.63, 1.77) | 1.51(0.96, 2.38) | 0.045 | 1.25(1.11, 1.39) |

Model 3: adjusted for age, sex, ischemia time, hypertension, hypercholesterolemia, diabetes, ASCVD, smoking status, eGFR, culprit vessel, multivessel lesion.
